# Risk factors and clinical presentation of craniocervical arterial dissection: A prospective study

**DOI:** 10.1186/1471-2474-13-164

**Published:** 2012-09-03

**Authors:** Lucy C Thomas, Darren A Rivett, John R Attia, Christopher R Levi

**Affiliations:** 1 Faculty of Health, The University of Newcastle, Callaghan 2308, New South Wales, Australia

## Abstract

**Background:**

Craniocervical arterial dissection is a major cause of ischaemic stroke in young adults. The pathogenesis is not fully understood but is thought to be related to a combination of an intrinsic weakness in the arterial wall and an external trigger. Intrinsic susceptibility is thought to be a generalised arteriopathy, vascular anomaly or genetic predisposition. Proposed extrinsic factors include recent viral infection and minor mechanical trauma to the neck, including neck manipulation, which has raised concerns amongst manual practitioners in particular as to the appropriate screening of patients and avoidance of more vigorous therapeutic techniques. The presenting features of dissection may mimic a musculoskeletal presentation, creating a diagnostic dilemma for primary care practitioners. Early recognition is critical so that appropriate management can be commenced.

The aims of this study are to prospectively investigate young patients ≤55 years admitted to hospital with radiologically diagnosed craniocervical arterial dissection compared to matched controls with stroke but not dissection, to identify risk factors and early presenting clinical features, so these may be more readily identified by primary care practitioners.

**Methods:**

Patients ≤ 55 years presenting to hospital with craniocervical arterial dissection and controls will have their medical records reviewed and be interviewed and questioned about possible risk factors, preceding events to admission such as recent neck trauma, and presenting clinical features including any preceding transient ischaemic features. Clinical assessment will include a connective tissue screening examination to identify subclinical connective tissue disorders. Radiology and blood screening will be reviewed for typical features and inflammatory markers. Functional outcome will be reviewed to determine the burden of the stroke.

**Discussion:**

This study will provide descriptive and comparative data on intrinsic and extrinsic risk factors for craniocervical arterial dissection and outline the typical clinical presentation, including the nature of early presenting features which might assist practitioners to identify those patients for whom vigorous manual therapy of the neck is inappropriate and alert them to those for whom immediate urgent medical care should be sought.

## Background

Craniocervical artery dissection (CAD), a tearing of the intimal, medial or adventitial layers of the wall of the internal carotid or vertebral arteries, is a major cause of ischaemic stroke in young to middle aged individuals in the fourth and fifth decades [1,2], perhaps accounting for up to 10-25 % of ischaemic stroke in this age group [3]. CAD has an annual incidence of 2.5-3:100,000 [3-5], however this may be an underestimate as cases with mild clinical signs and symptoms may not always be recognised and dissections may resolve spontaneously [2].

The aetiology of CAD is not fully understood and many cases are described as occurring spontaneously when no obvious mechanism or trigger can be identified. However, it has been suggested that for dissection to occur there is a contribution from both intrinsic and extrinsic factors. It has been proposed that the mechanism involves both a pre-existing intrinsic susceptibility such as an underlying arteriopathy, and a precipitating event which may be fairly innocuous [2,3,6,7]. The underlying arteriopathy may be in the form of a vascular anomaly, a genetic pre-disposition [8] such as a subclinical connective tissue disease, or may be a transient situation perhaps caused by an infection or pro-inflammatory state giving rise to a temporary friability of the vessel wall [9,10]. In patients with such an existing susceptibility, exposure to a precipitating event such as minor mechanical trauma or activities imparting some stress to the neck [11,12] may trigger a dissection of the artery [13]. Such minor trauma is usually innocuous, such as might occur during the course of normal daily activities. Frank trauma, such as may happen during a motor vehicle accident has not usually been reported [14,15].

Cervical spine manual therapy has been hypothesised as one type of minor trauma or neck stress which may be a trigger for CAD. This has raised concerns amongst manual practitioners as to whether the nature of manipulative techniques is responsible for reported cases or whether some patients already have CAD in its early stages when it is difficult to diagnose. Notably, the clinical presentation of CAD usually includes neck pain and headache, which may in many cases mimic a musculoskeletal disorder or migraine [2,3,7]. It is therefore possible that CAD might not be recognised early in its presentation, particularly in the absence of clear ischaemic (or neurological) features. This may lead the patient to seek pain treatment from their primary care practitioner or manual therapist for the painful symptoms which are in reality resulting from a dissection in progress [16].

Previous reviews have often included a number of retrospective studies of CAD [2,3,17] some of which have shown conflicting findings in respect of the presence of particular risk factors [2]. Exposure to minor mechanical trauma of the neck has been shown to be associated with CAD by a number of authors [4,13,18,19], however detailed characterisation of the types of trauma and direction of force application has been limited. Retrospective studies are often limited and biased by the information available in hospital databases, highlighting the need for prospective studies which include interviews of patients close to the time of their dissection.

Previous prospective studies have tended to be from large hospital series and were therefore medically focussed, again relying on routine information in the hospital records and did not always use a face to face interview [13-15,20,21]. They may have therefore been subject to selection bias if investigators were able to choose whether or not to include participants based on information available in the records [15,22]. Some previous studies have also lacked age limitations so may have included older patients with atherosclerosis or other age related conditions. Moreover, they did not always include a control group for comparison. In particular, they generally report limited information or historical details about preceding activities and events, as well as occurrence of any transient ischaemic features in the weeks preceding hospitalisation which might facilitate early recognition of CAD. These details are of particular interest to primary care practitioners to assist them to more readily identify those patients at risk of CAD or who may be presenting with early symptoms.

It has been argued that the timely recognition of potential risk factors and more subtle early presenting neurological signs or symptoms of CAD is critical [2,23] so that the patient is not exposed to inappropriate manual treatment of the neck. Early recognition is also critical in the case of a patient presenting with a dissection in progress, so that referral for appropriate medical management can be made promptly. There is also a need to characterise the presenting features of vertebral and internal carotid artery dissection, in particular ischaemic features, with more descriptive detail, so as to aid recognition of the significance of early signs and symptoms.

In manual therapy texts and guidelines on the topic of vertebrobasilar insufficiency (VBI) [24], an insufficiency of blood flow to the hindbrain, much emphasis has been placed on the presence of dizziness as an indicator of VBI. However it is possible that other clinical features may be earlier or more useful indicators of the presence of dissection and associated VBI. Identification of early clinical features related to dissection of craniocervical arteries may help in the prompt recognition of these conditions in patients presenting to physiotherapists and other practitioners.

A recent retrospective study of risk factors and clinical features of CAD by our research group examined the medical records of 47 patients ≤ 55 years who had suffered a vertebral or internal carotid dissection, and found that 64 % had a recent history of minor mechanical trauma to the neck [25]. Other preceding events and proposed risk factors such as recent infection and hypertension were less well documented. This retrospective study was however limited by inconsistent recording of data in the medical records. Hence we have designed a prospective study to further investigate the risk factors and presenting clinical features of CAD patients in the Hunter region of New South Wales, Australia.

The purpose of the proposed study is therefore to prospectively investigate the presenting clinical features and pre-existing health status of CAD patients ≤ 55 years in order to identify risk factors and describe the common early clinical features.

### Aims and hypotheses

The specific aims of the study are to test the following hypotheses in a prospective cohort of patients with radiologically confirmed CAD:

1. That the following risk factors will be independently associated with vertebral or internal carotid arterial dissection:

· recent minor mechanical trauma to the head or neck

· cervical spine manual therapy, specifically high velocity thrust manipulation or end-range rotational mobilisation techniques or deep upper cervical soft tissue massage

· recent infection, febrile illness or clinical markers of pro − inflammatory states

2. That patients presenting with craniocervical arterial dissection pain will have no antecedent ischaemic neurological features.

## Methods

### Design

**Figure 1:**
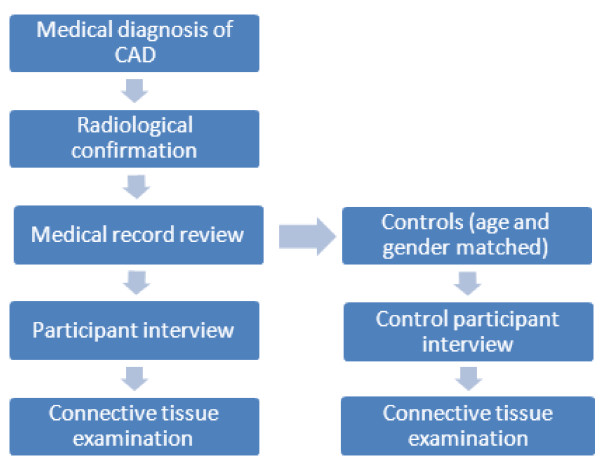
Participant recruitment and data collection.

The study is a prospective case control design examining patients with CAD and age and gender matched controls with ischaemic stroke but without CAD. The case–control design will be used to examine chronic risk factors, i.e. mild connective tissue disorder, vascular anomaly and cardiovascular factors, and clinical characteristics between participants. A case cross-over design will be used to examine acute risk factors or triggers i.e.; mechanical trauma and recent infection. Participants will be asked about exposure to the trigger event in the last 24 hours, the last week and within the last month. This will allow participants to act as their own controls as well as allowing comparison between dissection and control groups. The flow of participants through the study is shown in Figure 1.

### Participants

All patients ≤ 55 years presenting to hospital in the Hunter region of New South Wales, Australia with a radiological diagnosis of stroke caused by extracranial vertebral or internal carotid arterial dissection and who give their informed consent will be included in the study. Control participants will be age and gender matched patients who present with stroke from some other cause than dissection and who also give their informed consent to participate. Patients with CAD of iatrogenic origin will be excluded. Patients presenting with subarachnoid haemorrhage (SAH) will be excluded because this is a rare condition with distinctively different presenting features from dissection. Patients with primary intracranial dissection will also be excluded as this is commonly associated with SAH.

The diagnosis of CAD will be confirmed by radiological review. Imaging will be performed using computed tomography (CT) or magnetic resonance imaging (MRI), as ordered by the admitting neurologist. Radiological imaging will be reviewed online post hoc by two neurologists to describe the radiological features. Criteria applied will be visualisation of the following typical features of dissection [26]:

i). dissection flap or double lumen

ii). mural haematoma

iii). crescent sign – a crescentic rim of hyperintense signal

iv). long tapering stenosis characterised by string or ‘pearl and string’ sign

v). increase in external diameter or vertebral or internal carotid artery due to wall thickening or narrowing of the lumen

vi). pseudoaneurysm or dissecting aneurysm.

The degree of vessel stenosis, defined as a reduction in the lumen, will be graded as a percentage of vessel occlusion. Cerebral infarction will be defined as high signal on diffusion weighted imaging (B = 1,000) in the acute phase and as high signal on FLAIR T2 weighted imaging in the subacute phase. Infarct topography will be mapped. Imaging will also be inspected for any evidence of vascular variant or anomaly of the anterior or posterior cerebral circulation.

### Participant characteristics

Descriptive data will be collected including demographic details, the type and location of the dissection, the presence of infarction and the burden of stroke. This information will be collected from the patient records and by structured interview when the participant is medically stable. The interview will be undertaken by one of the investigators who is a registered physiotherapist with post-graduate qualifications in manual therapy and 28 years of clinical experience. Burden of stroke will be assessed at discharge from the discharge summary; details of residual signs and symptoms, Modified Rankin Score (mRS) [27] and National Institute of Health Stroke Score (NIHSS)[28]. The mRS is widely used for assessing global outcomes following stroke. The score is out of five where zero denotes full recovery, five denotes death and a score of two or less means a patient is able to walk and manage their own affairs [27]. The mRS has been demonstrated to have acceptable inter-rater reliability (κ =0.71-0.95) [27,29,30]. The NIHSS is a well validated tool used in the evaluation of neurological deficit in stroke patients [31]. It has moderate to excellent inter-rater reliability (ICC 0.82), and high validity (r = 0.68) when compared to infarct volume on CT imaging [31,32].

### Measurement of risk factors for CAD

Details of risk factors will be collected from the medical notes and a structured participant interview (see Additional file 1: Appendix 1). For the acute risk factors (minor mechanical trauma and recent infection) participants will be asked about exposure to these events within the last 24hours, the last week and the previous month.

#### Minor mechanical trauma

Minor mechanical trauma or stress to the neck will be investigated under the following categories based on the work of Dittrich et al. [13]:

· Heavy lifting

· Direct and indirect trauma to the head and neck

· Jerky or abrupt movements of the head

· Sporting activities

· Manual therapy.

If any trauma or stress to the neck is reported, specific descriptive details of the amount of force involved and direction of movement of the head and neck will be sought from the participant. If the participant has undergone recent manual therapy to the neck, descriptive details of the therapeutic procedure received will be sought from the participant.

#### Recent infection

Recent infection is defined as any infection or viral illness reported by the participant within the last month. Details of the type and severity of the condition, whether it was confirmed by a health professional and the need for medical intervention, such as antibiotic treatment, will be identified in the medical records or from the patient interview. Details of haematological results at the time of admission, specifically full blood count, erythrocyte sedimentation rate, C-reactive protein, immunological studies and coagulation times will be collected from the medical records. These will be examined for the presence of existing pro-inflammatory factors.

#### Vascular anomaly

Vascular anomaly or anatomical variant is defined as radiological evidence of a hypoplastic or aplastic cerebral artery or an anomalous course or termination of a cerebral artery such as a vertebral artery ending in posterior inferior cerebellar artery. Evidence of vascular anomaly or anatomical variant will be identified from the review of radiological imaging of the participants.

#### Cardiovascular factors

The presence of cardiovascular factors are defined as a reported medical diagnosis of hypertension, hypercholesterolemia, history of smoking, diabetes, migraine, family history of young stroke and contraceptive pill use.

· Hypertension is defined as a systolic pressure >140 mmHg and diastolic pressure >90 mmHg according to Australian Heart Foundation guidelines[33].

· Hypercholesterolemia is defined as a total cholesterol level >200 mg/dL[34]

· Smoking (current/past, cigarettes/day)

· Diabetes- (medical diagnosis of type one or type two diabetes)

· Migraine (medical diagnosis of migraine, on medication for migraine, self-report of migraine)

· Family history of young stroke <55 years

· Current or recent contraceptive pill use

#### Connective tissue disorder

Evidence of mild connective tissue disorder is defined as joint hypermobility, skin hyperextensibility or skin fragility. These features will be measured using a 25 item scale described by Dittrich et al. [35]. A sum score of all positive items will be calculated, where 0 = no disorder and scores >10 = strong disorder. The cut off used for the presence of connective tissue disorder is eight, based on the work of Dittrich [35]. The examination includes tests for joint hypermobility and skin fragility and extensibility. Also included will be additional questions about a reported history of hypermobile joints and the ability to contort the body as a child.

Joint hypermobility measurement includes items comprising the Beighton scale [36]. This has been shown to have good inter-rater reliability (ICC 0.72, 0.79) [37,38]. Skin extensibility is measured manually by pinching the skin on the volar aspect of the forearm one third of the distance from the elbow to the wrist with the elbow at 90° or in full extension. Skin is pulled up and the distance measured and quantified as ≤1 cm, >1 cm, >2 cm, >3 cm. >4 cm or >5 cm, with the pathological level determined as >2 cm [39].

### Measurement of presenting clinical features of CAD

Detailed characterisation of the features that commonly present during the process of both vertebral and internal carotid artery dissections will be collected. In addition the presence of any early warning signs, notably transient ischaemic signs or symptoms in the preceding month will be collected. Features will be categorised under the following headings:

· Headache (presence, location, severity)

· Neck pain (presence, location, severity)

· Facial palsy (presence of ptosis/Horner’s syndrome)

· Visual disturbance (presence of blurred vision, diplopia, hemianopia)

· Speech disturbance (presence of dysarthria, dysphonia, dysphasia such as expressive or receptive)

· Balance disturbance (presence of dizziness, imbalance, unsteadiness, falls)

· Paraesthesia (presence, location such as upper limb/lower limb, face)

· Weakness (presence, location such as upper limb/lower limb).

Questions about clinical features not expected to be present will also be included as distractors. These questions will be about the presence of chest pain and cognition. Information will be initially sought in the medical records and additional details, including the time frame of onset of signs and symptoms, obtained from a structured patient interview (see Additional file 1: Appendix 1). The interview will be undertaken by a member of the research team as soon as the participant is medically stable.

### Statistical analysis

The projected sample size is 100 dissection patients and 100 controls. The sample size is based on ten subjects per prognostic indicator, based on a review of the literature [2,18,25] Indicators evaluated will include mechanical trauma, recent infection, vascular anomaly, hypertension, hypercholesterolemia, smoking, diabetes, migraine, family history and oral contraception.

Demographic data, risk factors and outcome will be analysed using descriptive and comparative statistics. Risk factors will be analysed using simple logistic regression to generate odds ratios and p values. All factors with a p value > 0.2 will be included in a multiple regression model with outcomes expressed as odds ratios with 95 % confidence intervals. Statistical analysis will be performed with STATA statistical analysis software (version 11, Statacorp, Texas, USA).

Ethical approval for the study has been granted by the Hunter New England Human Research Ethics Committee.

## Discussion

The proposed study will help to further define risk factors for CAD, in comparison to a control group, in a young population in whom CAD is most commonly described. The prospective nature of the study and the use of a structured interview will allow more detailed analysis of the preceding events and presenting clinical signs and symptoms than has previously been undertaken. Quantifying the burden of stroke for both dissection and non-dissection stroke patients may help inform decisions concerning the provision and extent of post-acute rehabilitative care.

In the case of recent minor mechanical trauma to the neck, the study may allow more detailed information to be gained on the nature of the trauma, forces involved and direction of movement of the neck. This may be helpful to identify typical traumatic events, such as a blow to the head or neck during a sporting or other commonly undertaken activity, which might be implicated in the onset of dissection.

The detailed characterisation of presenting clinical features of dissection in this prospective study may help to assist primary care practitioners to more readily identify this serious pathology in patients who present seeking conservative pain relief. Descriptive details of radiological features and infarct topography may assist better recognition of vertebral and internal carotid artery dissection in young stroke patients admitted to emergency departments.

Similarly, if mechanical trauma or infection or other risk factors are shown to be associated with CAD in this study, this might be useful to raise the index of suspicion amongst primary care practitioners of the potential diagnosis of CAD in the young to middle aged patient for example, who presents with sudden onset of headache and or neck pain and a recent history of mechanical trauma to the head or neck. This may help to both avoid inappropriate treatment in such people and prompt further investigation, potentially expediting medical intervention, in the case of a suspected dissection.

## Competing interests

The authors declare no conflict of interest.

## Authors’ contributions

All authors were involved in the design of the study. All authors read and approved the final manuscript.

## Funding

The study forms part of the doctoral studies of Lucy Thomas and is not supported by any external funding.

## Pre-publication history

The pre-publication history for this paper can be accessed here:

http://www.biomedcentral.com/1471-2474/13/164/prepub

## Supplementary Material

Additional file 1 Appendix 1. Guide for patient interview regarding clinical features and possible risk factors. Click here for file
