# Supplementary material for: Risk factors and clinical presentation of craniocervical arterial dissection: A prospective study
Source: BMC Musculoskelet Disord. 2012 Sep 3;13:164. doi: 10.1186/1471-2474-13-164 (PMC3441544; doi:10.1186/1471-2474-13-164)

**Craniocervical arterial dissection study**

**Interview guide**

**Introduction**

Hello, I’m *name*. I’m a researcher with the stroke team with Dr *name*. We are doing some research into stroke in young people and I would like to ask you some questions.

Can you tell me what happened when you had your stroke? What symptoms prompted you to seek help? *(List)*

**Clinical features**

1. **Headache**

Did you have a headache? Can you show me where you felt it *(record on body chart)?*

Can you describe how it felt? *(descriptive details e.g. throbbing, thunder clap, sharp, ache)*

How severe was the pain? If I asked you to score the pain severity out of ten, where zero is no pain and ten the worst pain you can imagine, where would you score this pain?

Have you ever experienced this pain before?

How long did the pain last?

1. **Neck pain**

Did you have neck pain? Can you show me where it was *(record on body chart)*?

What did it feel like? How bad was the pain /10?

1. **Chest pain/ shortness of breath**

Did you have any chest pain?

Did you have any palpitations?

Were you short of breath?

1. **Facial palsy**

Did you notice any drooping of one side of your mouth or one eyelid?

Did you notice one eye weeping?

Did you notice any unusual feelings on your face or tongue such as pain or pins and needles?

1. **Visual disturbance**

Did you notice any change in your vision such as blurring or double vision, or that parts of your vision were not there?

1. **Speech disturbance**

Did you notice any difficulty with your speech such as a hoarse voice, difficulty speaking, slurred speech, difficulty with expression - finding words or losing words when speaking?

Did you find yourself muddling words or using the wrong word when you were speaking?

1. **Balance disturbance**

Did you experience any dizziness or loss of balance?

Can you describe the feeling? (*descriptive details e.g. spinning of self or room, floating, sea sickness, vague imbalance)*

Did this make you feel nauseous or vomit?

Did you notice you were off balance or veering to one side when you were standing or walking?

Did you have any falls? Do you know what caused you to fall?

1. **Paraesthesia/ anaesthesia**

Did you notice any numbness, pins and needles or tingling in your arms or legs?

Can you show me where it was *(body chart)*?

1. **Weakness**

Did you notice any weakness in your arms or legs? For example, have you noticed your leg giving way or have you tripped or dropped things or found it difficult to pick up small objects?

Did you have any of these symptoms before your stroke lasting only a short time *(record nature and duration)*?

1. **Cognition**

Did you notice any change in your mental function?

Were you confused?

Did you have difficulty understanding people?

**Risk factors**

1. **Recent minor mechanical trauma**

In the last 4 weeks have you done any of the following (*record details and time frame prior to stroke onset)*:

Heavy lifting over 25 kg with maximum effort?

Suffered a blow to your head or neck?

Suffered an indirect blow to your head or neck such as a mild whiplash injury?

Activities involving jerky head movement where you had to move your head suddenly?

Unusually vigorous sports activity? e.g. jogging for more than 45 mins

Any manual treatment of your neck such as manipulation, forceful neck turning, or deep massage to the top of your neck.

Have you ever had any adverse reaction to this type of treatment in the past?

Control period: Did you have any injury to your head or neck in the 24 hours prior to coming into hospital, on the same day in the previous week, or the same day one month before *(eg if hospital admission is on a Wednesday, the previous Wednesday and Wednesday 4 weeks prior)*

1. **Recent infection**

In the last 4 weeks have you had any infection or viral illness such as the flu or a gastric illness? (*record details and time frame prior to stroke onset)*

How long ago was this?

Did you see a doctor for this and what was the treatment?

Control period: Did you have any infection of viral illness in the 24 hours prior to coming into hospital,on the same day in the previous week, or the same day one month before *(eg if hospital admission is on a Wednesday, the previous Wednesday and Wednesday 4 weeks prior)*

1. **Cardiovascular**

What is your general health like? Are you usually healthy?

Do you have any of the following?

- High blood pressure? Are you receiving treatment for this?
- High cholesterol? Are you on medication for this?
- Diabetes? Are you receiving treatment for this?
- Diagnosed migraine? Are you on medication for this?

Do you smoke? How many cigarettes a day?

Has anyone in your family had a stroke under 55 years of age?

Are you currently taking the contraceptive pill?

**Body Chart**

Record area of pain or paraesthesia or anaesthesia


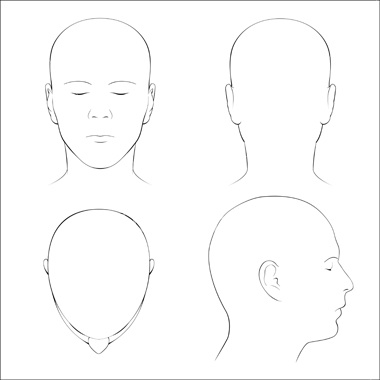

Supplement: Additional file 1 — Appendix 1. Guide for patient interview regarding clinical features and possible risk factors. [file 1471-2474-13-164-S1.docx]
